# Machine learning and bioinformatics identify and validate membrane protein biomarkers for type 2 diabetes mellitus

**DOI:** 10.3389/fendo.2026.1805548

**Published:** 2026-07-24

**Authors:** Tong Liu, Zhaoyuan Tang, Zulipikaer Aierken, Jie Zhang, Cheng Lei

**Affiliations:** 1 State Key Laboratory of Pathogenesis, Prevention and Treatment of Central Asian High Incidence Diseases, Clinical Medical Research Institute, The First Affiliated Hospital of Xinjiang Medical University, Urumqi, Xinjiang, China; 2 Basic Medical College, Xinjiang Medical University, Urumqi, Xinjiang, China; 3 The 3rd Affiliated Teaching Hospital of Xinjiang Medical University (Affiliated Cancer Hospital), Urumqi, Xinjiang, China

**Keywords:** biomarker genes, diagnostic model, differentially expressed genes, membrane protein-related genes, type 2 diabetes mellitus

## Abstract

**Introduction:**

Type 2 diabetes mellitus (T2DM) is among the most rapidly increasing metabolic disorders worldwide. Membrane proteins, integral components of biological membranes, are pivotal in insulin signal transduction and significantly contribute to the pathogenesis of T2DM. However, the systematic investigation of membrane proteins linked to T2DM remains insufficient.

**Methods:**

The T2DM dataset and membrane protein-related genes were sourced from the GEO database and the Uniprot website, respectively. Bioinformatics methodologies, including Gene Ontology (GO) analysis, Kyoto Encyclopedia of Genes and Genomes (KEGG) pathway enrichment analysis, and protein-protein interaction (PPI) network analysis, were employed to assess the differentially expressed membrane protein-related genes between the normal control group and the T2DM group. Subsequently, machine learning algorithms, including Gaussian Mixture Model (GMM), Random Forest (RF), and Support Vector Machine (SVM), were utilized to identify hub genes. Following this, a clinical diagnostic model was constructed, and the receiver operating characteristic (ROC) curve was plotted. Candidate genes were further examined in palmitic acid (PA)-induced NES2Y and HepG2 cell models and in high-fat diet/streptozotocin-induced T2DM mice. Finally, Functional effects were assessed by gene knockdown, reverse transcription quantitative PCR (RT-qPCR), western blotting (WB), immunofluorescence (IF), co-immunoprecipitation, histological examination, and lipid staining.

**Results:**

A total of 1,856 DEGs were identified between T2DM and control samples, including 599 upregulated genes and 1,257 downregulated genes, among which 42 were membrane proteinrelated T2DM DEGs. Machine learning algorithms were then applied to pinpoint two target genes: ICAM1 and EZR, the latter of which encodes Ezrin, a membrane–cytoskeleton linker protein. The ROC curve analysis showed that the diagnostic model exhibited strong predictive capability, with an AUC value of 0.95. PA treatment increased lipid accumulation and EZR/ICAM1 mRNA and Ezrin/ICAM1 protein expression in the cell models. Ezrin co-immunoprecipitated with the insulin receptor. In PA-treated HepG2 cells, ICAM1 or EZR knockdown increased p85α and AKT phosphorylation and GLUT4 protein expression. Ezrin and ICAM1 were also elevated in the livers of T2DM mice.

**Discussion:**

ICAM1 and EZR may serve as potential diagnostic biomarkers and candidate therapeutic targets associated with T2DM. Furthermore, ICAM1 and EZR may be associated with insulin resistance by reducing glucose transport efficiency, potentially through modulation of the PI3K-AKT signaling pathway.

## Introduction

Diabetes mellitus is a chronic metabolic disorder marked by persistently elevated blood glucose levels, a condition referred to as hyperglycemia. It is primarily classified into type 1 diabetes and type 2 diabetes mellitus (T2DM) (1). Over 90% of diabetes cases are attributed to T2DM, a chronic metabolic disorder characterized by insulin resistance leading to hyperglycemia, impaired insulin secretion, or insufficient compensatory insulin secretion responses (2). According to the 10th edition of the International Diabetes Federation report, the global prevalence of T2DM among individuals aged 20 to 79 years was estimated to be 10.5% (536.6 million people) as of 2021. This prevalence is projected to rise to 783.2 million by 2045 (3). T2DM is associated with various environmental and genetic risk factors, exhibiting significant heterogeneity among patients (4). Understanding the biological pathways that contribute to the pathogenesis of T2DM and its complications, particularly those related to the characteristics of membrane proteins, is crucial for early diagnosis, personalized treatment, and disease prevention (5–7). Consequently, the identification of novel screening biomarkers and therapeutic targets has become a focal point in current T2DM research.

Membrane proteins are crucial components of biological membranes, playing a key role in various cellular processes, such as cell adhesion, signal transduction, ion accumulation, and energy transfer (8). Recent studies have demonstrated that membrane proteins are integral to the pathogenesis and progression of T2DM: (1) modulating the translocation and activity of glucose transporters, thereby influencing cellular glucose uptake; (2) participating in insulin receptor signaling transduction, which affects the downstream PI3K/Akt pathway; (3) mediating the exchange of lipids and glucose metabolites between the cell membrane and intracellular organelles, indirectly regulating insulin sensitivity. These mechanisms collectively maintain glucose homeostasis, and their dysfunction can lead to insulin resistance (9). The mechanisms by which proteins such as G protein-coupled receptors (GPCRs) (10), solute carrier family members (such as SLC30A8) (11), and ion channels (such as TRPV1) (12) contribute to energy metabolism and cellular communication are not yet well understood. Despite the growing recognition of the importance of membrane proteins in diabetes, systematic screening of membrane proteins associated with T2DM remains limited. There is an urgent need to adopt innovative methodologies for the identification and validation of these essential genes (13).

The comprehensive analysis of transcriptomic data from diverse databases has become a pivotal method for identifying novel differentially expressed genes (DEGs) and uncovering their biological roles in the context of disease (14). Currently, high-throughput proteomics, bioinformatics analysis, and targeted mass spectrometry can effectively identify and validate membrane protein biomarkers associated with T2DM (15, 16). In this study, we utilized bioinformatics techniques alongside machine learning strategies to elucidate the relationship between genes associated with membrane proteins and T2DM. Additionally, we developed a predictive model for T2DM. Importantly, *in vitro* and *in vivo* experiments validated the functional roles of the key molecules EZR, which encodes Ezrin, a membrane-cytoskeleton linker protein, and ICAM1 in T2DM. Our findings may contribute to the identification of diagnostic biomarkers for T2DM and aid in the development of more effective therapeutic interventions.

## Materials and methods

### Data collection

**Figure 1:**
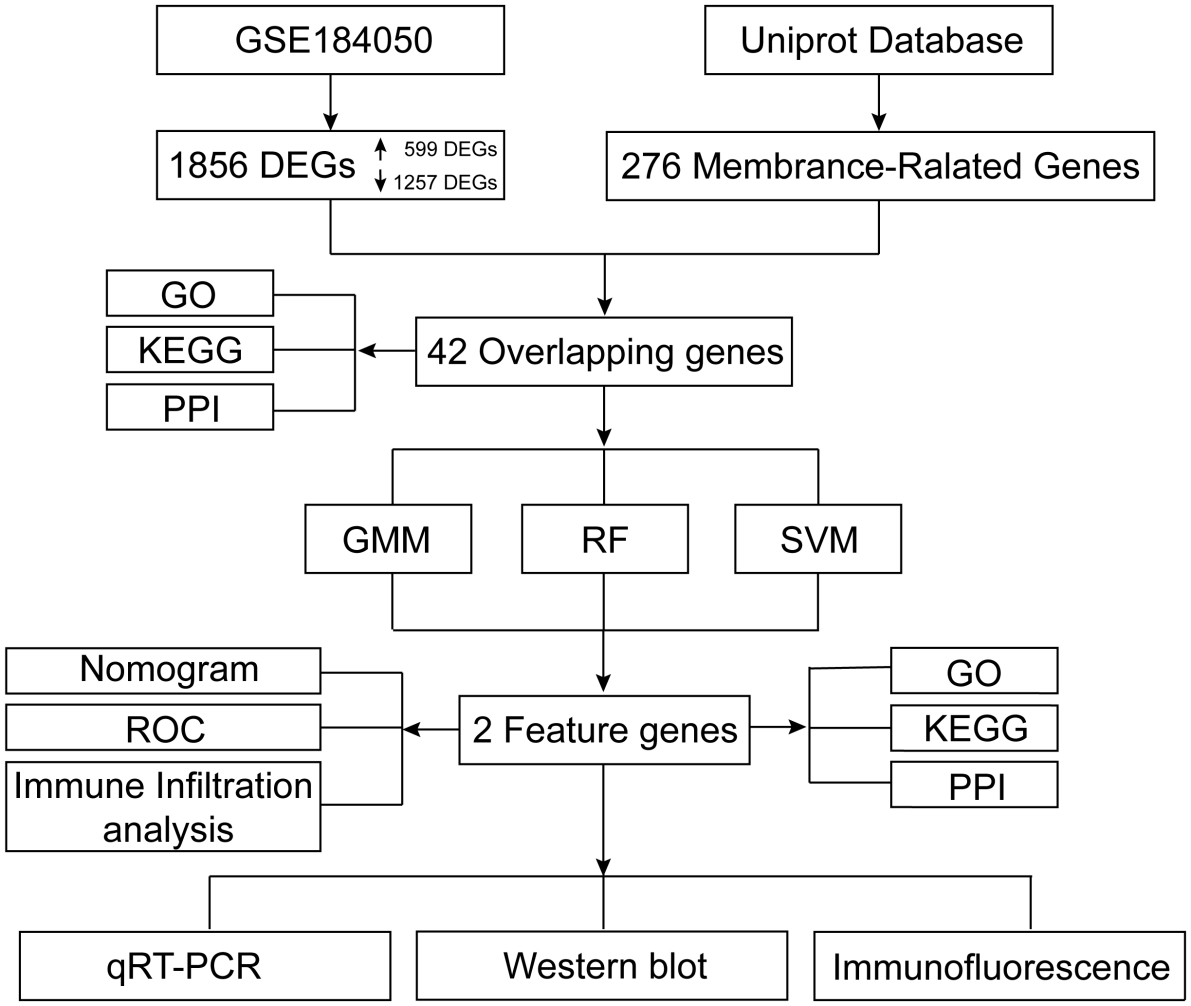
Workflow of the analysis.

The flowchart of our data analysis pipeline is illustrated in **Figure 1**. The microarray data for the GSE184050 dataset were obtained from the GEO database (https://www.ncbi.nlm.nih.gov/geo/), comprising samples from 50 individuals with type 2 diabetes mellitus and 66 healthy controls. Subsequently, we used advanced search on the UniProt protein database (https://www.uniprot.org/), searched for “membrane rafts” in subcellular localization terms, and then selected the species as human, resulting in a total of 276 associated genes (17, 18).

### Screening and analysis of DEGs

Prior to analysis, principal component analysis (PCA) was employed to evaluate the distribution of normal islet samples and T2DM islet samples. To enhance data quality, low-quality samples were excluded based on PCA results (19, 20). Additionally, the normalized gene expression data from dataset GSE184050 was assessed to determine the relative consistency of gene expression across individual islet samples, thereby validating the comparability between insulin samples and normal samples. Differential expression analysis of normal and T2DM islet samples was conducted using the “Limma” package within Bioconductor (21). To control for multiple testing, *p* values were adjusted using the Benjamini-Hochberg false discovery rate (FDR) method (22). Genes with an FDR-adjusted *p* < 0.05 and |log2FC| > 1 were defined as DEGs. Genes with log2FC > 1 were considered upregulated, whereas those with log2FC < -1 were regarded as downregulated. Volcano plots and bubble charts were generated utilizing an online bioinformatics analysis platform (https://www.bioinformatics.com.cn). Venn diagrams were created using the online Venn diagram tool Venny2.1 (https://bioinfogp.cnb.csic.es/tools/venny/) to identify DEGs associated with membrane protein.

### Gene ontology and Kyoto encyclopedia of genes and genomes pathway enrichment analysis

To explore the functions and interactions of these membrane protein-related DEGs in biological pathways, we employed the STRING online database for GO and KEGG pathway enrichment analysis (23). GO and KEGG enrichment analyses were performed to explore the biological functions and pathways associated with membrane protein-related DEGs. GO, and KEGG analysis was conducted by employing gene set variation analysis (GSVA) and gene set enrichment analysis (GSEA) with the R package ‘GSVA’ and ‘clusterProfiler’, respectively (24, 25). Furthermore, GO enrichment analysis was supplemented using the Metascape online tool (https://metascape.org) (26).

### Construction of the protein-protein interaction network for DEGs

The PPI network was constructed using the STRING database (available at http://string-db.org) to predict protein-protein interactions and elucidate the overarching organizing principles of functional cellular systems (23). Using the following criteria: degree cutoff = 2, node score cutoff = 0.2, and k-core = 2, confidence score cutoff = 0.7 and maximum depth=100 (27). The PPI network was analyzed and visualized using the Maximum Neighborhood Component (MNC) method through the CytoHubba plugin of Cytoscape software (Version 3.7.2). To enhance network reliability, nodes with low connectivity were excluded, and the top 10 genes ranked by MNC score were designated as hub genes. The resultant network underwent further validation through functional enrichment and survival analyses.

### Machine learning

To identify candidate biomarkers and establish diagnostic models, we employed three machine learning algorithms: Gaussian Mixture Model (GMM), Random Forest (RF), and Support Vector Machine (SVM). To reduce the risk of overfitting and avoid information leakage, the GSE184050 dataset was used as the discovery cohort and was randomly divided into a training set and an internal testing set using stratified sampling, thereby preserving the proportion of T2DM and control samples in both subsets. Feature selection was performed only in the training set. GMM, RF, and SVM were then applied to screen candidate diagnostic genes, and cross-validation was used during model construction to optimize model parameters and evaluate model stability. Genes identified at the intersection of these three algorithms are considered the core diagnostic genes (28–30).

### Construction of nomograms and assessment of receiver operating characteristic curves

The selected feature genes were used to construct a diagnostic nomogram with the “rms” R package to predict the probability of T2DM. ROC curve analysis was performed to evaluate the diagnostic performance of the selected feature genes and the nomogram, and the area under the curve (AUC) was used as the metric of diagnostic accuracy. Model performance was first assessed in the training set and internal testing set, and independent GEO datasets were further used for external validation to evaluate the generalizability of the diagnostic model.

### GeneMANIA database

GeneMANIA (http://genemania.org/) is a functional association database that possesses robust capabilities for retrieving data related to input genes. This includes information on physical interactions, co-expression, co-localization, predictions, gene enrichment analysis, genetic interactions, shared protein domains, and pathways.

### The correlation between hub genes and diabetes

The correlation between hub genes and diabetes was analyzed using the Attie Lab Diabetes database (http://diabetes.wisc.edu). This database provides a searchable resource for gene expression data, allowing users to examine the gene expression profiles of various experimental groups (C57BL/6 obese mice at 4 weeks and 10 weeks of age, as well as BTBR obese mice) in any of six tissues, including liver (31).

### Palmitic acid induces insulin resistance in human hepatoma HepG2 cells

HepG2 cells were obtained from Meisen Chinese Tissue Culture Collections (MeisenCTCC, China) and cultured in DMEM (HyClone, USA) supplemented with 10% FBS (ExCellBio, China) at 37 °C in a 5% CO_2_ atmosphere. To establish a PA (H8780, Solarbio, China) induced IR model in HepG2 cells, the cells were incubated with 0.5 mM PA for 24 hours. The control group was treated with vehicle control (BSA without fatty acids, containing 2.5%). Each experimental condition was performed in triplicate.

### Cell viability assessment

The effect of different concentrations of PA on the viability of HepG2 cells was assessed using the Cell Counting Kit-8 (CCK8) assay (BS350B, Biosharp, China). Briefly, cells were seeded in a 96-well plate and incubated for 24 hours, followed by treatment with varying concentrations of PA for an additional 24 hours. After the treatment, CCK8 solution was added and incubated for 1 hour. The optical density (OD) was measured at a wavelength of 450 nm. The cell viability of the control group was set to 100%. Each group was conducted in six replicates and repeated three times.

### Glucose consumption experiment

HepG2 cells were seeded in a 96-well plate, with six wells designated as blank controls. After a 24-hour incubation, the cells were exposed to various concentrations of PA for an additional 24 hours. Following treatment, the culture medium was removed, and the cells were washed twice with PBS. The medium was then replaced with DMEM supplemented with 0.5% FBS and containing 11.1 mmol/L glucose. After another 24-hour incubation, the culture medium was collected, and glucose consumption was calculated using a glucose assay kit (BTK013, Bioswamp, China) by subtracting the glucose concentration in the experimental wells from that in the blank wells.

### Reverse transcription quantitative PCR

We conducted RT-qPCR to quantify the expression of EZR and ICAM1 in human pancreatic β-cell line NES2Y and HepG2 cells, both with and without PA treatment. RNA was extracted from the cells using TRIzol reagent (Invitrogen, 15596-026), and subsequently reverse transcribed into complementary DNA (cDNA) according to the manufacturer’s instructions using the Hiscript II QRT Supermix qPCR kit (Vazyme, China). qPCR was performed using the ChamQ Universal SYBR kit (Vazyme, China) on the Bio-Rad CFX96 real-time PCR detection system, with the thermal cycling conditions set to 95 °C for 2 minutes, followed by 35 cycles of 95 °C for 10 seconds and 60 °C for 30 seconds. β-actin was utilized as an internal reference gene. The relative mRNA expression was calculated using the 2−ΔΔCt method, where ΔΔCt = (Ct, target gene − Ct, reference gene) − (Ct, target gene − Ct, reference gene) in the control. The experiments were performed in triplicate. All primer sequences were designed using Primer3plus (https://www.primer3plus.com) (**Table 1**).

**Table 1:** Primer sequences used in RT-qPCR.

Genes	Forward (5’-3’)	Reverse (5’-3’)
EZR	TGGCCCTCGTAGCCTTGAGGAC	CCAGTGCTGCAGGGTCCGAGGT
ICAM1	GTCACCTATGGCAACGACTCCTTC	AGTGTCTCCTGGCTCTGGTTCC
β-actin	CCACCATGTACCCAGGCATT	CGGACTCATCGTACTCCTGC

### Plasmid construction and transfection

The knockdown plasmids for EZR and ICAM1 were pLV3-U6-EZR (human)-shRNA-Puro, pLV3-U6-ICAM1 (human)-shRNA-Puro, and their corresponding negative control vectors, constructed by Miaoling Biotechnology (Wuhan, China). HepG2 cells were seeded in 24-well plates at 30%-40% confluency and transfected with the plasmids using polyethyleneimine (PEI; C0541-1ml, Beyotime, China) as the transfection reagent. After 48 hours of transfection, drug screening was performed with 200μg/ml G418 (ST081, Beyotime, China) and 10 μg/ml puromycin (ST551, Beyotime, China) respectively. The transfection efficiency was verified by western blot.

### Western blotting

Protein expression was analyzed using western blotting. Cells or liver tissues were lysed in RIPA buffer containing protease and phosphatase inhibitors, and total protein concentrations were determined using a BCA protein assay. Equal amounts of protein were loaded in each lane and separated by SDS-PAGE, followed by transfer onto PVDF membranes. The membrane was subsequently blocked and incubated with primary antibodies: ICAM1 (ER131207, HUABIO, China, 1:1,000), Ezrin (222624, Zenbio, China, 1:1,000), INSR (R381357, Zenbio, China, 1:1,000). GAPDH (#AF7021, Affinity, China, 1:10,000) and β-actin (A5316, Sigma-Aldrich, Germany, 1:25,000) were used as loading controls. Following washing with TBST (G2150-1L, Servicebio, China), the membrane was treated with HRP-conjugated secondary antibody rabbit IgG (4030-04, Southern Biotech, USA), and protein bands were detected using an enhanced chemiluminescence system.

For quantitative analysis, band intensities were measured using ImageJ software. After background subtraction, the gray value of each target protein band was normalized to the corresponding loading control. For phosphorylated proteins, the band intensity was normalized to the corresponding total protein, including p-p85α/p85α and p-AKT/AKT. The normalized values were then expressed relative to the control group, which was set to 1. Quantitative data were obtained from independent biological replicates and are presented as mean ± SD.

### Co-immunoprecipitation assay

The cells were lysed in 200 µl of RIPA buffer (R0010, Solarbio, China) and incubated overnight at 4 °C with a primary antibody. Subsequently, the cell lysate (0.4 ml) was incubated with protein A/G magnetic beads (30 µl) (B23202, Selleck, USA) conjugated with the antibody at 4 °C for 3 hours. After centrifugation at 800 × g for 5 min at 4 °C, the immunoprecipitates separated by magnetic beads were washed five times with RIPA lysis buffer and boiled with sample loading buffer. The resulting immunoprecipitates were subjected to immunoblotting analysis as previously described. The primary and secondary antibodies used for Co-IP were consistent with those used in the western blot experiments.

### Immunofluorescent staining

After treating HepG2 cells with PA for 24 hours, the cells were fixed at room temperature with 4% paraformaldehyde for 15 minutes, followed by three washes with PBS, each lasting 5 minutes. The fixed cells were then incubated in 0.1% Saponin (84510, Sigma, USA) in PBS for 30 minutes, and subsequently washed three times with PBS. The cells were blocked with 5% BSA (Bovine Serum Albumin) (SW3015, Solarbio, China) for 30 minutes, followed by three washes with PBS, each for 5 minutes. The cells were then incubated at room temperature with a specific primary antibody for 2 hours, after which they were washed four times with PBS, each for 5 minutes, and incubated with goat anti-rabbit or anti-mouse secondary antibody at room temperature for 1 hour. The procedure for the second protein in the co-localization immunofluorescence experiment was identical to that described above. Finally, the cells were stained with DAPI (BL105A, Biosharp, China) for 5 minutes, followed by three washes with PBS, each for 5 minutes, and the fluorescent images were visualized using a confocal microscope (Nikon AXR).

### Animal experiments

All animal studies were approved by the Animal Experiment Ethics Review Committee of Xinjiang Medical University. Male C57BL/6 mice (6 weeks old) were purchased from the Animal Center of Xinjiang Medical University (Urumqi, China). Each group in the studies comprised of six mice. To induce T2DM model in C57BL/6 mice, twelve mice were randomly assigned to two groups, with six mice in each group. The control group was fed a standard diet, while the experimental group received a high-fat diet (HFD, 60% fat) for a duration of five weeks. In the sixth week, the mice on the high-fat diet underwent intraperitoneal injection (for five consecutive days at a dosage of 50 mg/kg body weight; dissolved in a 0.1 M citrate buffer, pH 4.5) to further induce the T2DM model (32). Following twelve weeks of HFD feeding, the liver tissues of the mice were subjected to subsequent histopathological examination and western blot analysis.

### Histopathological analysis

Liver tissue was fixed in 4% formaldehyde, embedded in paraffin, and sectioned into slices with a thickness of 4 μm. Subsequently, these slices underwent deparaffinization and were stained with hematoxylin-eosin (H&E) for general histological observation. The stained liver tissue sections were imaged using a panoramic slide scanner (3DHISTECH CaseViewer, Pannoramic SCAN, Hungary). Image analysis was conducted using CaseViewer v2.4 software, capturing images at magnifications ranging from 100× to 400×.

### Oil Red O staining

Lipid staining was performed using an Oil Red O staining kit (G1262, Solarbio, China), following the manufacturer’s instructions. Stained cells were imaged using an optical microscope (CX31, Olympus, Japan).

### Immunohistochemical analysis

After sacrifice, mouse liver tissues were collected, excised, and fixed in 4% paraformaldehyde. The embedded liver tissues were sectioned for histological analysis. Following dewaxing, hydration, and antigen retrieval procedures, the sections were incubated with 10% goat serum (AR1009, Boster, China) at room temperature for 20 minutes. Subsequently, the sections were subjected to immunostaining with anti-ICAM1 antibody (ER131207, HUABIO, China, 1:200) and anti-Ezrin antibody (222624, Zenbio, China, 1:100) at 4 °C overnight. Following this, the sections were incubated with the secondary antibody (PV-9000, ZSGB-BIO, China) at 37 °C for 60 minutes, and finally, the color development was performed using a DAB color development kit (ZLI-9018, ZSGB-BIO, China), with IHC images captured under a microscope.

### Statistical analysis

Experimental data were analyzed using GraphPad Prism 9.0 software (GraphPad Prism Software Inc., CA, USA). Data were expressed as mean ± standard deviation (SD). For cell experiments, n = 3 represents three independent biological replicates. For animal experiments, n = 6 mice per group were used. Statistical analyses were performed using unpaired t test for comparisons between two groups and one-way analysis of variance (ANOVA) for comparisons among multiple groups. For transcriptomic differential expression analysis, multiple testing correction was performed using the Benjamini-Hochberg FDR method. An FDR-adjusted *p* < 0.05 was considered statistically significant for DEG screening.

## Results

### Screening for DEG between normal and T2DM insulin samples

**Figure 2:**
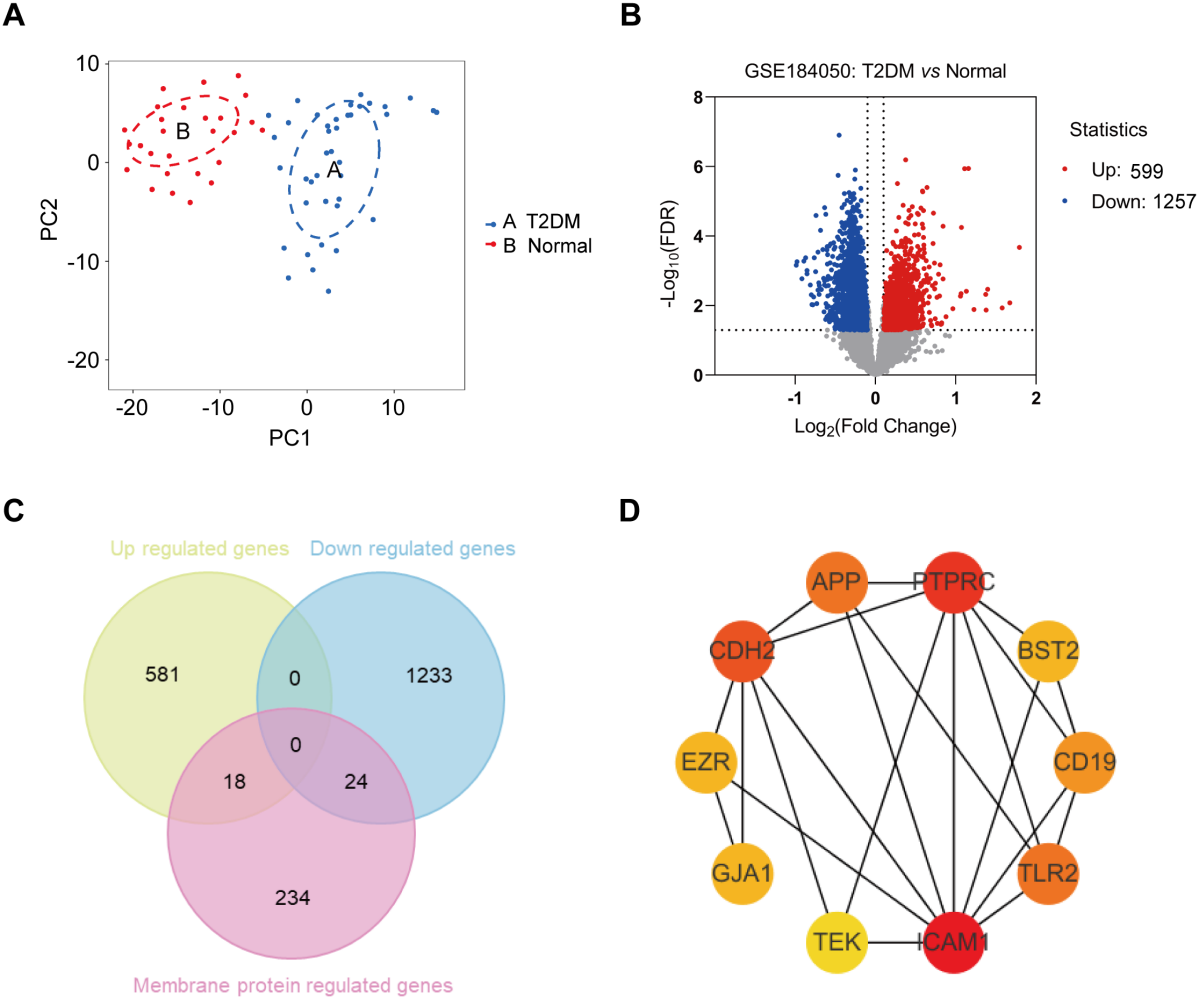
Screening for differential genes. **(A)** PCA of normal pancreatic islet samples and T2DM pancreatic islet samples. **(B)** Volcano plot illustrating differentially expressed genes, where blue represents downregulated genes, red indicates upregulated genes, and black denotes genes under non-significant conditions. **(C)** Venn diagram showing the intersection of up regulated genes, down regulated genes, and membrane protein regulated DEGs. **(D)** The PPI network shows the interactions of the top10 genes and the edges type is co-expression.

**Figure 3:**
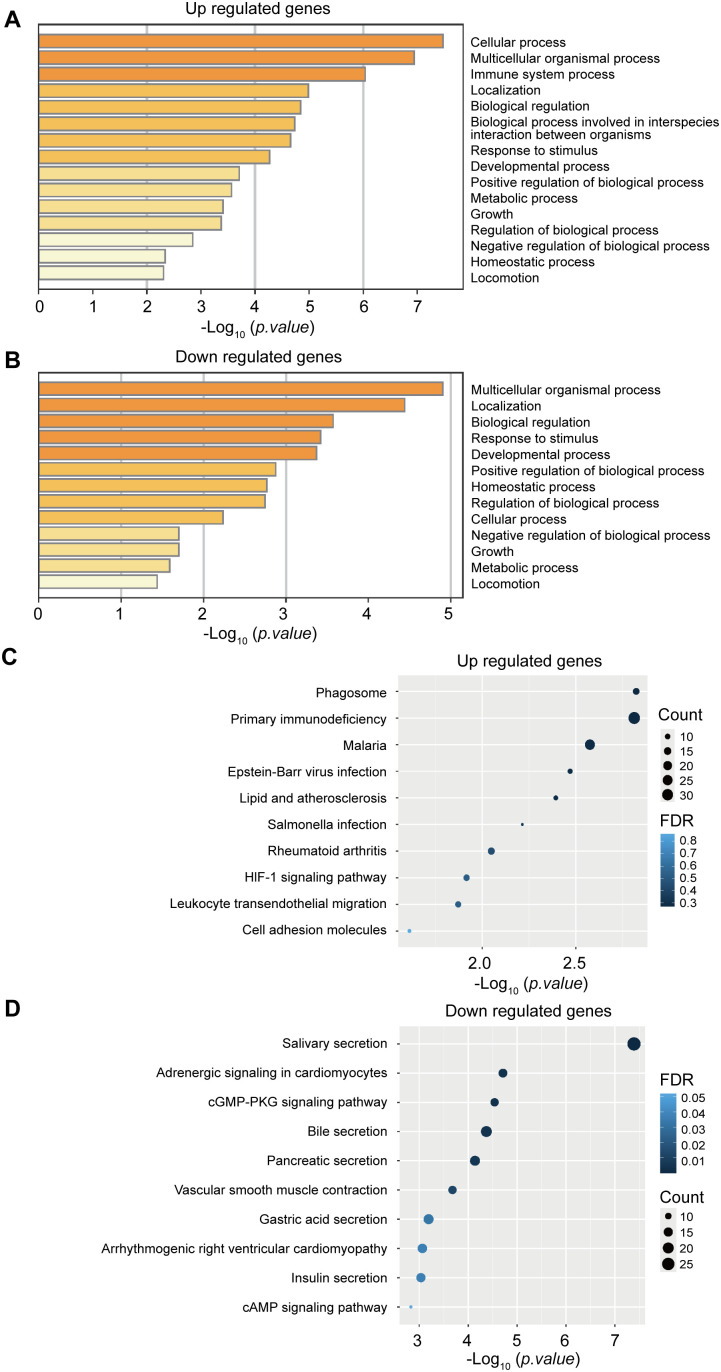
Functional enrichment analysis of characteristic genes. **(A, B)** GO analyses of up and down regulated genes. **(C, D)** KEGG analyses of up and down regulated genes.

To investigate the potential mechanisms underlying the onset of T2DM, we retrieved microarray data from the GEO database (GSE184050), comprising 50 T2DM samples and 66 healthy control samples. We initially conducted PCA to ascertain whether there were discernible expression patterns between the T2DM and control groups (**Figure 2A**; **Supplementary Table 1**). Visualization through a volcano plot revealed a total of 1,856 DEGs within the GSE184050 dataset, including 599 upregulated genes and 1,257 downregulated genes (|log2FC| > 1 and FDR-adjusted *p* < 0.05, **Figure 2B**) (33, 34). By intersecting the membrane protein gene dataset with the DEGs from the T2DM dataset, we identified 42 common genes associated with membrane proteins and T2DM DEGs (**Figure 2C**; **Supplementary Table 2**). The STRING database was employed to investigate the PPI of 42 membrane protein regulated DEGs. Using the MNC algorithm from the CytoHubba plugin (35), 10 candidate hub genes were identified within the PPI network: ICAM1, PTPRC, CDH2, TLR2, APP, CD19, ABCG2, GJA1, TEK, EZR (**Figure 2D**). We utilized the Metascape tool to analyze the involvement of 18 upregulated genes and 24 downregulated genes in disease pathways. For the upregulated genes, GO enrichment analysis indicated significant involvement in pathways related to immune system processes, biological regulation, and metabolic processes (**Figure 3A**). KEGG pathway analysis demonstrated that the differential genes were primarily enriched in the Phagosome, Primary Immunodeficiency, and Lipid and Atherosclerosis pathways (**Figure 3C**). In the case of downregulated genes, GO enrichment analysis revealed a predominant association with multicellular organismal processes and biological regulation (**Figure 3B**). KEGG pathway analysis indicated that the differential genes were mainly enriched in Pancreatic secretion and Insulin secretion pathways (**Figure 3D**).

### Selection of feature genes using multiple machine learning algorithms

**Figure 4:**
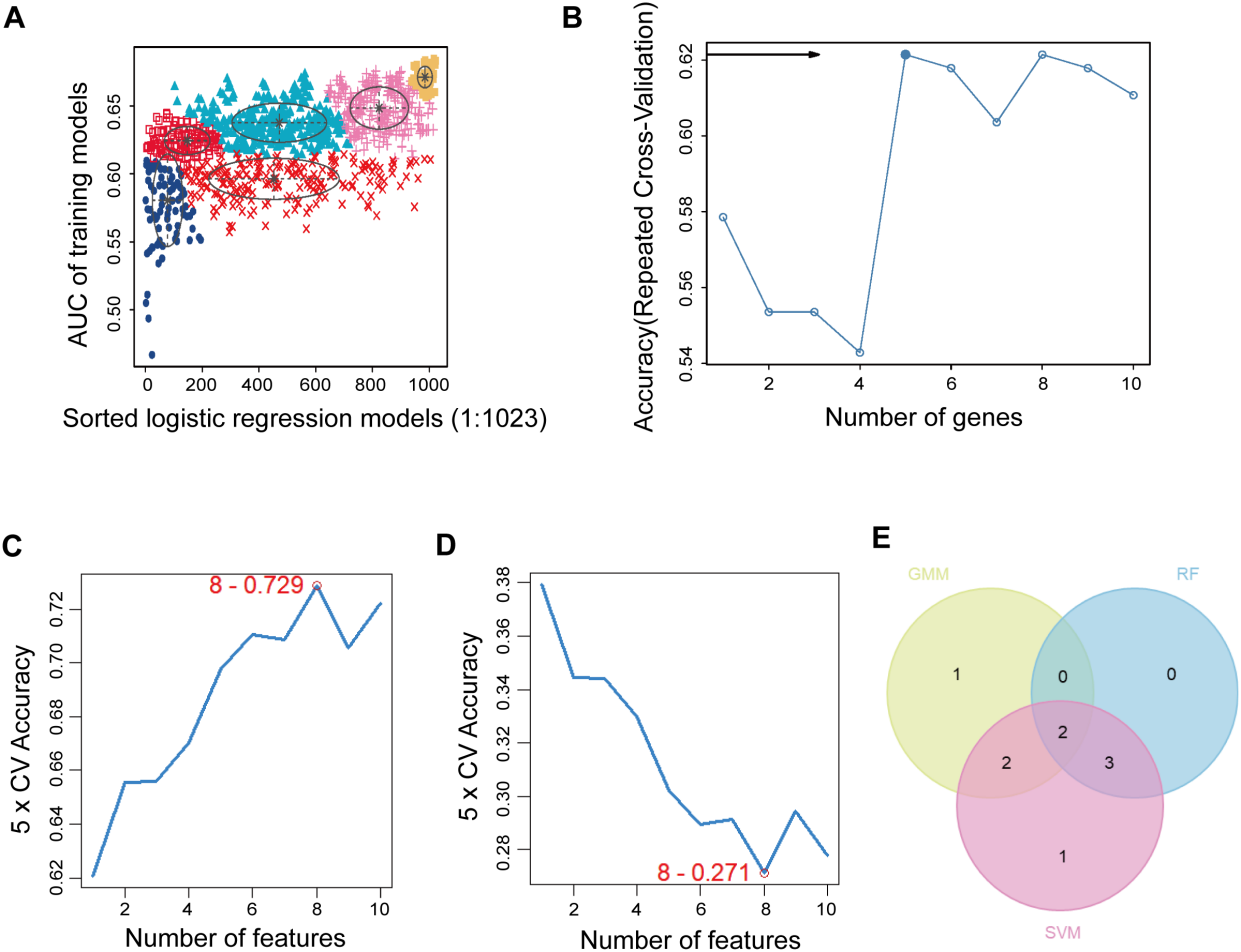
Machine learning identifies differentially expressed genes to membrane proteins. **(A)** Key genes identified in the Gaussian Mixture Model. **(B)** The Random Forest algorithm screens the key genes. **(C, D)** Support Vector Machine screens differentially expressed genes. **(E)** Venn diagram displayed the overlap of genes screened by three machine learning algorithms.

Using the predicted results of PPI, we identified ten intersecting genes between T2DM and membrane proteins to explore potential diagnostic biomarkers. In order to minimize the overfitting phenomenon, we conducted feature selection based on machine learning in the training set, and evaluated the stability of the model through cross-validation. Ultimately, two disease-associated differentially expressed genes were identified. Based on these ten genes, various bioinformatics methods were employed to further screen for diagnostic markers. Utilizing the Gaussian Mixture Model (GMM) algorithm, five genes were selected as potential biomarkers (**Figure 4A**). The Random Forest (RF) algorithm identified five candidate genes (**Figure 4B**). The Support Vector Machine (SVM) analysis demonstrated that when the number of eigengenes was set to eight, the highest accuracy and the lowest error rate were achieved, with an accuracy of 0.729 and an error rate of 0.271 (**Figures 4C, D**). Finally, through the intersection of the three algorithms, EZR and ICAM1 were identified as diagnostic biomarkers for T2DM (**Figure 4E**; **Supplementary Table 3**).

### Construction of a diagnostic model for T2DM based on membrane protein-associated genes

**Figure 5:**
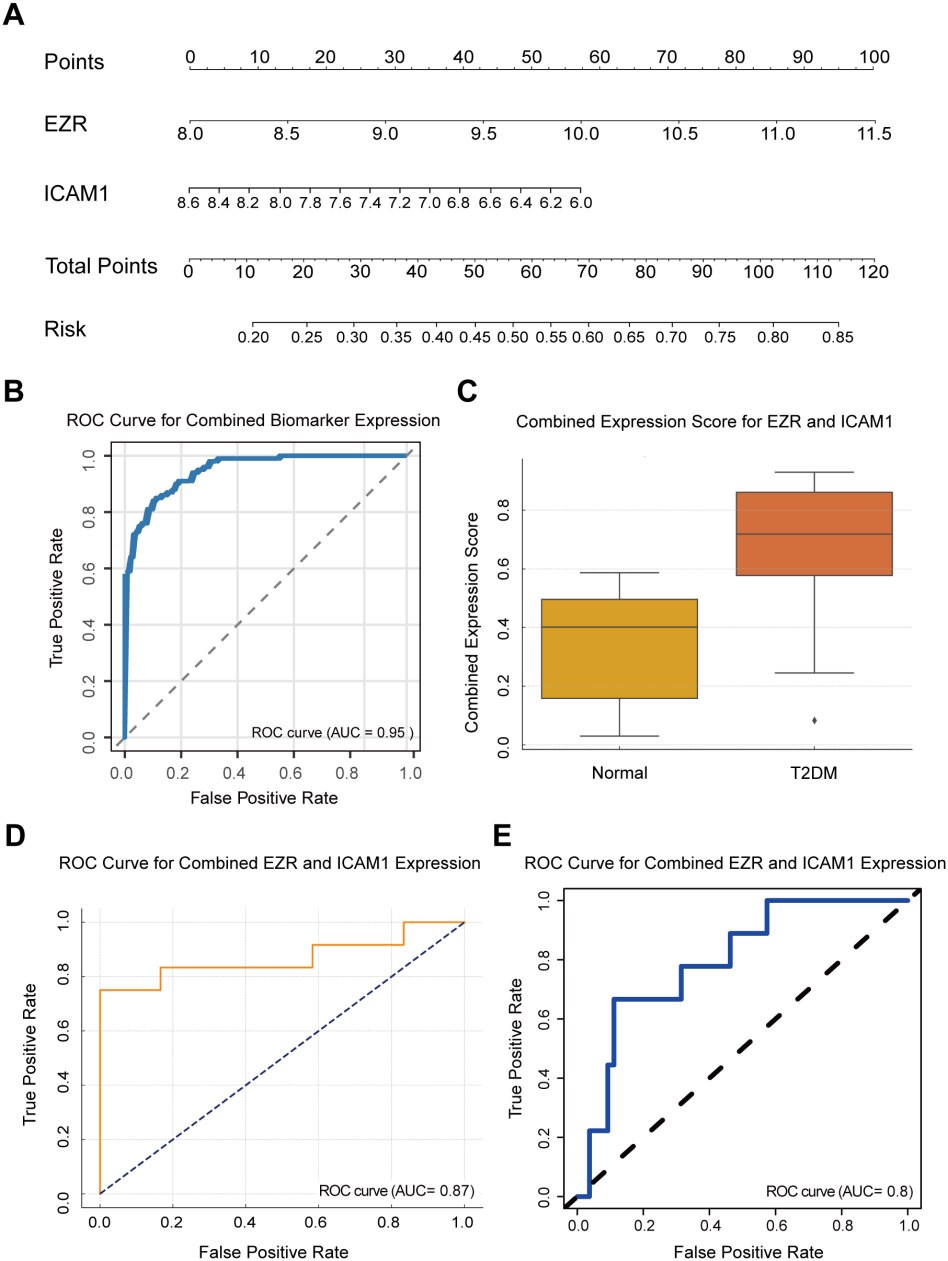
Construction and validation of the nomogram model for T2DM diagnosis. **(A)** A nomogram was used to predict the incidence of T2DM. **(B)** ROC curves analysis of the feature genes in the training dataset (GSE184050). **(C)** EZR and ICAM1 were highly expressed in the T2DM group of independent datasets (GSE29221). **(D, E)** ROC curves of the feature genes in the external validation datasets GSE29221 and GSE38642.

We utilized the “rms” package to construct a nomogram and employed calibration curves to assess its predictive capability for the probability of T2DM (**Figure 5A**). To mitigate the risk of overfitting, ROC curve analyses were conducted on both the training and testing datasets, and cross-validation was employed during the model evaluation process. The diagnostic model based on ICAM1 and EZR demonstrated excellent diagnostic performance, with an AUC of 0.95 in the GSE184050 dataset (**Figure 5B**). The expression levels and diagnostic efficacy of EZR and ICAM1 were further validated using independent external datasets, including GSE29221 and GSE38642 (**Figures 5C–E**). These findings suggest that the model possesses promising diagnostic potential for type 2 diabetes.

### Functional enrichment analysis of characteristic genes

**Figure 6:**
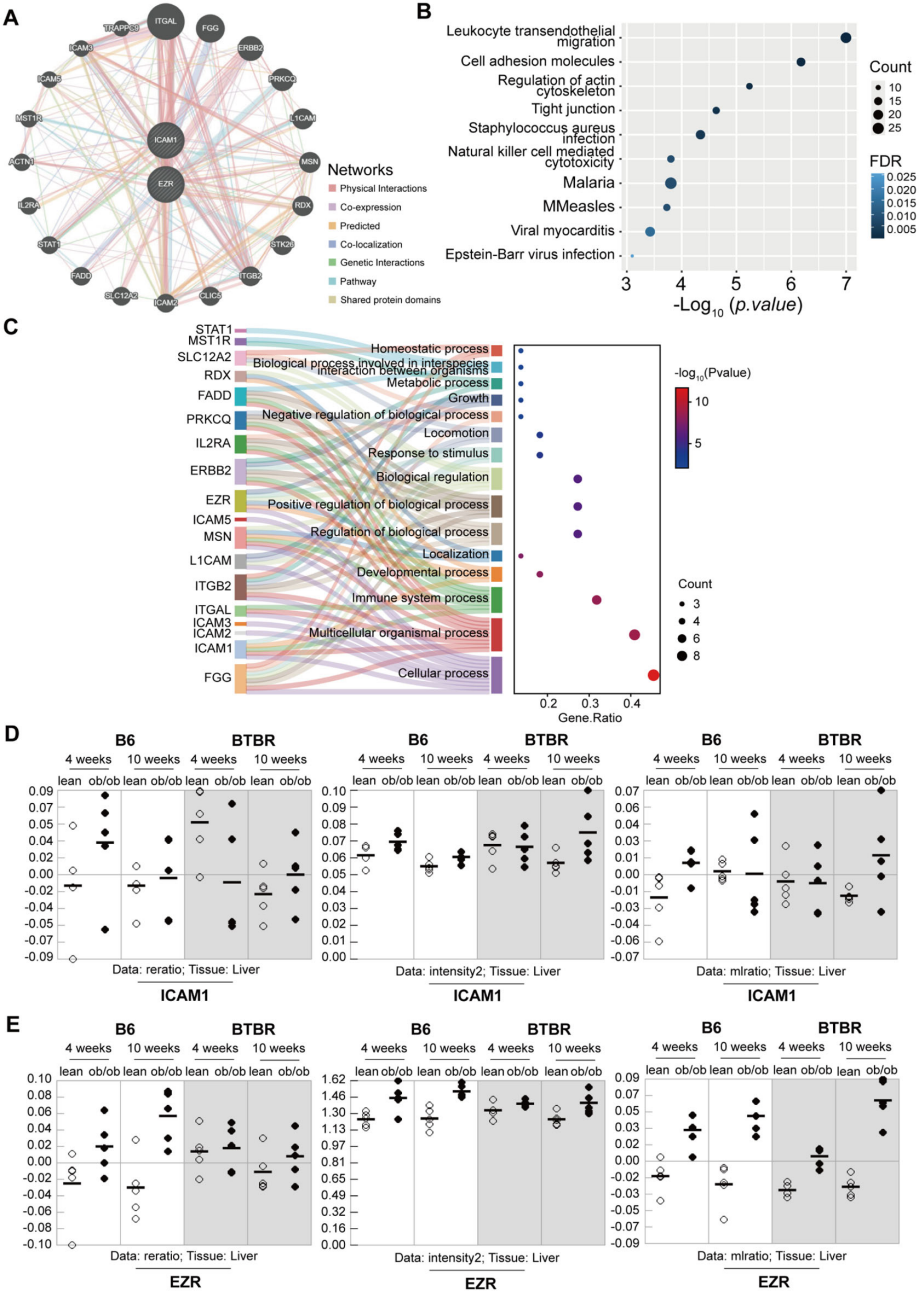
Functional enrichment analysis of ICAM1 and EZR and validation of mouse datasets. **(A)** The co-expression network of ICAM1 and EZR. **(B, C)** GO enrichment analysis of characteristic genes. **(D, E)** Validation of ICAM1 and EZR upregulation in liver tissues from 10-week-old obese diabetic mice.

A protein-protein interaction network of feature genes was established using the GeneMANIA database, identifying 20 genes that interact with EZR and ICAM1 (**Figure 6A**). Subsequently, GO and KEGG enrichment analyses were conducted on these 22 genes utilizing the Metascape database. The GO enrichment analysis of the 22 genes revealed that they were mainly involved in the regulation of biological and metabolic process (**Figure 6B**). The GO enrichment analysis of the 22 genes revealed that they were mainly involved in the regulation of biological and metabolic process (**Figure 6C**). Finally, to explore the correlation between these hub genes and diabetes, the Attie Lab Diabetes database was employed. C57BL/6 and BTBR mice, which develop severe diabetes with obesity at 10 weeks of age, were used for analysis. Through the reratio, intensity2 and mlratio algorithms, we identified a significant upregulation of ICAM1 and EZR expression in the 10-week-old C57BL/6 and BTBR obese diabetic mice, indicating a correlation with diabetes (**Figures 6D, E**).

### Effects of different concentrations of PA on viability and glucose consumption of HepG2 cells

**Figure 7:**
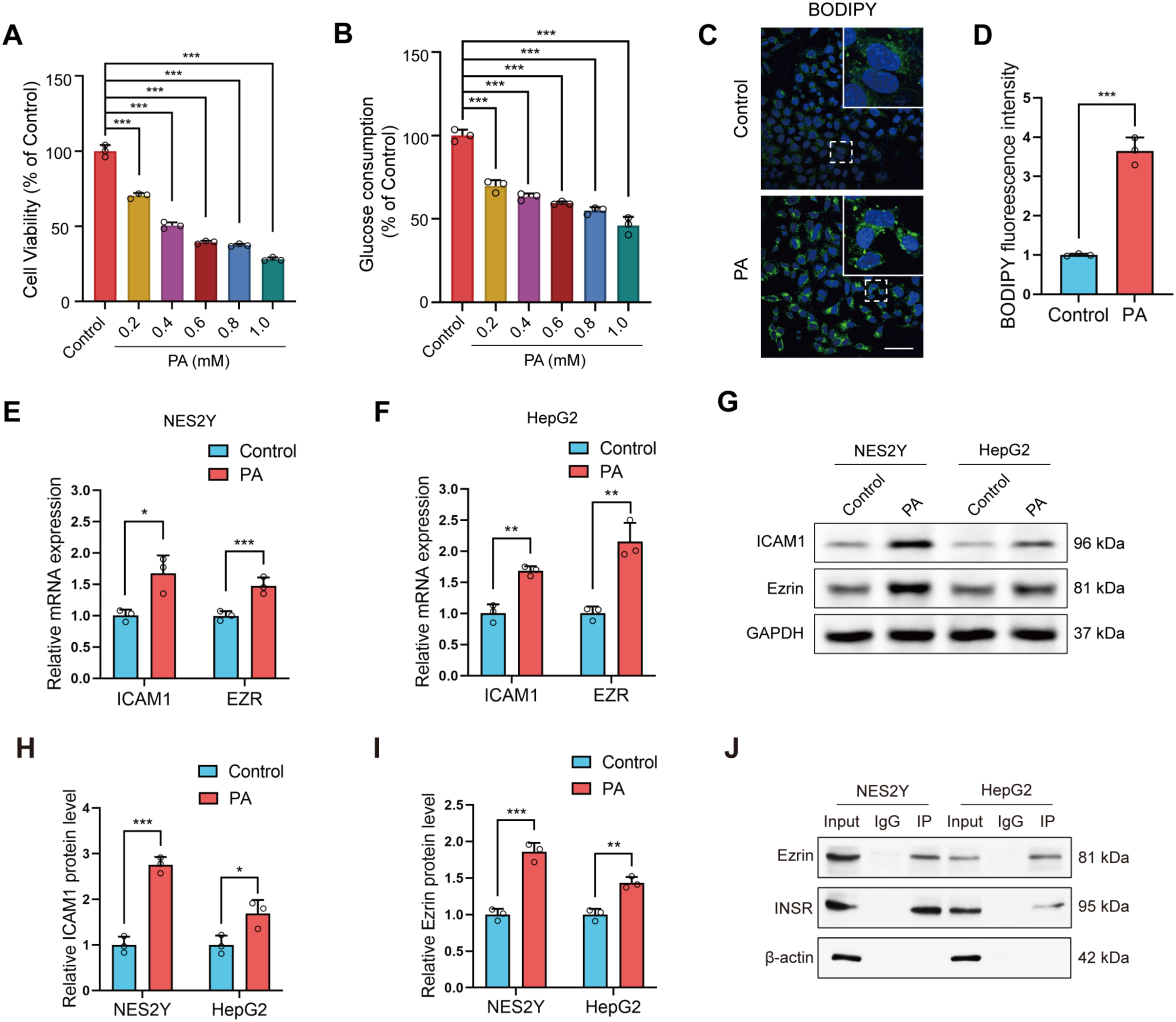
RT-qPCR and western blotting validation of EZR/ICAM1 mRNA and Ezrin/ICAM1 protein in NES2Y and HepG2 cells treated with PA. **(A)** CCK8 assay was performed to examine the viability of HepG2 cells exposed to indicated concentrations of PA for 24 h. **(B)** Glucose consumption was measured in HepG2 cells exposed to indicated concentrations of PA for 24 h. **(C)** BODIPY staining reveals the accumulation of lipid droplets. Scale bar, 50 μm. **(D)** Statistical analysis of fluorescence intensity of BODIPY stained lipid droplets. **(E)** The relative mRNA expression levels of EZR and ICAM1 in human islet cells treated with PA compared to the control group. **(F)** The relative mRNA expression levels of EZR and ICAM1 in human liver cancer cells treated with PA compared to the control group. **(G)** The protein expression level of ICAM1 and Ezrin after treatment with PA in NE2SY and HepG2 cells. **(H)** Protein expression statistics of ICAM1. **(I)** Protein expression statistics of Ezrin. **(J)** The Co-IP experiment confirmed the interaction between Ezrin and INSR in NES2Y and HepG2. The data are expressed as mean ± SD. n = 3 per group. *p < 0.05, **p < 0.01, ***p < 0.001 *vs* control group.

Excessive free fatty acids in obese patients contribute to the onset and progression of insulin resistance (36, 37). PA, a saturated fatty acid and a major component of free fatty acids, is commonly utilized to induce insulin resistance in HepG2 cells, resulting in a reduction of glucose consumption *in vitro* (38). Results from the CCK8 assay indicated that treatment with PA for 24 hours led to a dose-dependent decrease in cell viability (*p* < 0.01) (**Figure 7A**) and glucose consumption (*p* < 0.01) (**Figure 7B**). In HepG2 cells, the half-maximal inhibitory concentration (IC50) was approximately 0.5 mM. Consequently, in subsequent experiments, a concentration of 0.5 mM PA was selected to establish the insulin resistance model in HepG2 cells.

### The expression of EZR/ICAM1 mRNA and Ezrin/ICAM1 protein in the PA-induced insulin resistance model of HepG2 cells

We conducted BODIPY staining in HepG2 cells to observe the extent of lipid droplet accumulation. We found that, compared to the control group, the accumulation of lipid droplets was significantly increased following PA induction (**Figures 7C, D**). Subsequently, we assessed the expression levels of EZR and ICAM1 in NES2Y and HepG2 cells, with and without PA treatment, using RT-qPCR. Compared to the control group, there was a significant upregulation of ICAM1 and EZR expression in both NES2Y and HepG2 cells (**Figures 7E, F**). Subsequently, western blotting analysis demonstrated that PA treatment markedly increased the protein levels of ICAM1 and Ezrin in both NES2Y and HepG2 cell lines (**Figures 7G–I**). To investigate whether Ezrin, as a plasma membrane protein, directly participates in the regulation of insulin signaling, we performed Co-IP assays and demonstrated a direct interaction between Ezrin and insulin receptor (INSR) in both NES2Y and HepG2 cell (**Figure 7J**).

### The localization and expression of Ezrin and ICAM1 in NES2Y and HepG2 cells

**Figure 8:**
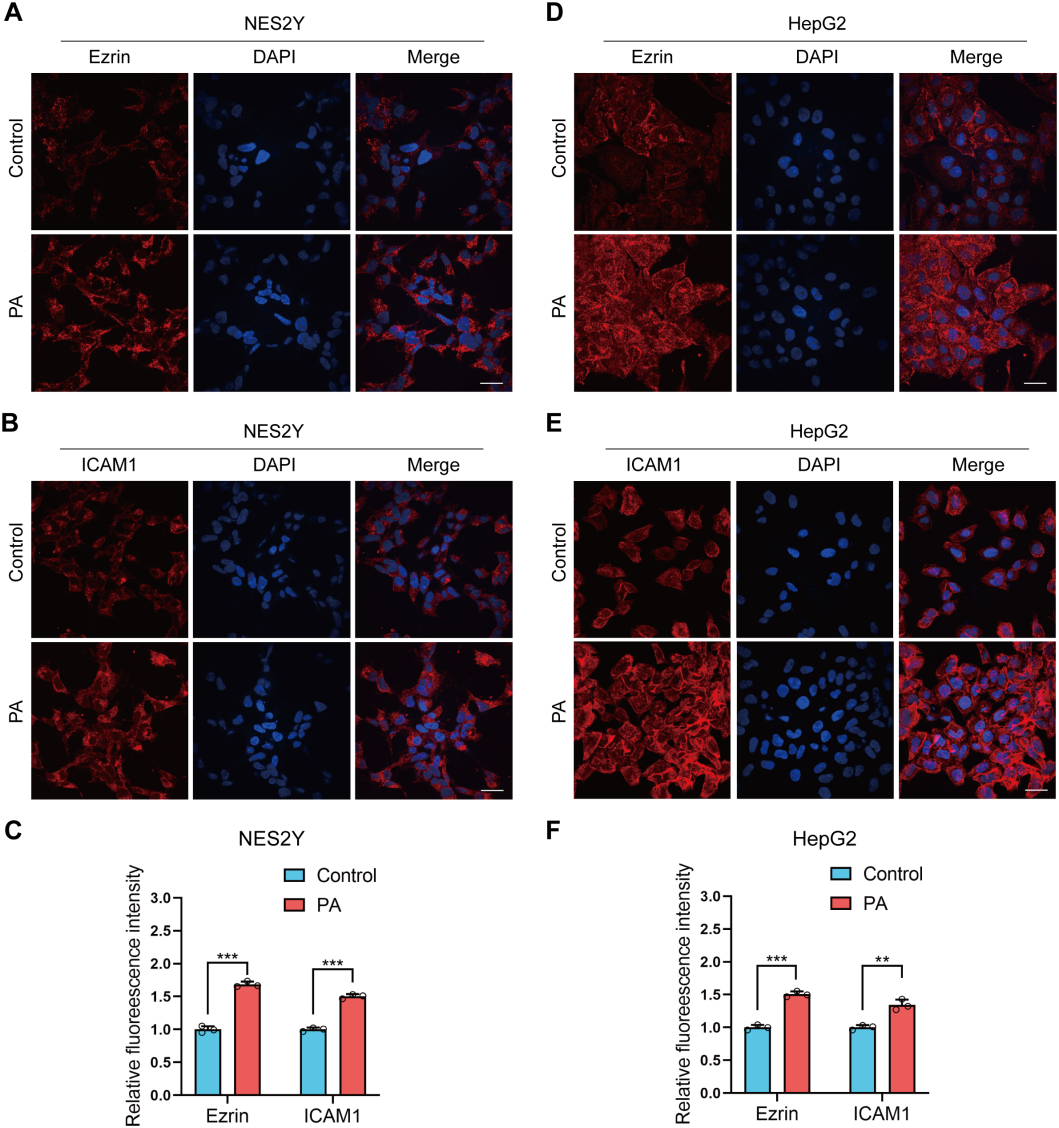
Immunofluorescence validation of the localization of Ezrin and ICAM1 in NES2Y and HepG2 cells. **(A, B)** Immunofluorescence staining showing the intracellular localization and fluorescence intensity of Ezrin and ICAM1. Scale bar, 25 μm. **(C)** Relative fluorescence intensity of Ezrin and ICAM1 in NES2Y cells. **(D, E)** Immunofluorescence staining showing the intracellular localization and fluorescence intensity of Ezrin and ICAM1 in HepG2 cells. Scale bar, 25 μm. **(F)** Relative fluorescence intensity of Ezrin and ICAM1 in HepG2 cells. The data are expressed as mean ± SD. n = 3 per group. **p < 0.01, ***p < 0.001 *vs* control group.

Through immunofluorescence staining, we observed a significant increase in the fluorescence intensity of Ezrin and ICAM1 in NES2Y cells compared to the control group (**Figures 8A–C**), as well as a notable increase in the fluorescence intensity of Ezrin and ICAM1 in HepG2 cells relative to the control group (**Figures 8D–F**). Ezrin and ICAM1 exhibited prominent localization on the cell membrane of HepG2 cells, and following PA induction, the membrane localization of Ezrin and ICAM1 was markedly enhanced, with an increase in fluorescence intensity.

### Knockdown of Ezrin and ICAM1 activates the PI3K/AKT signaling pathway

**Figure 9:**
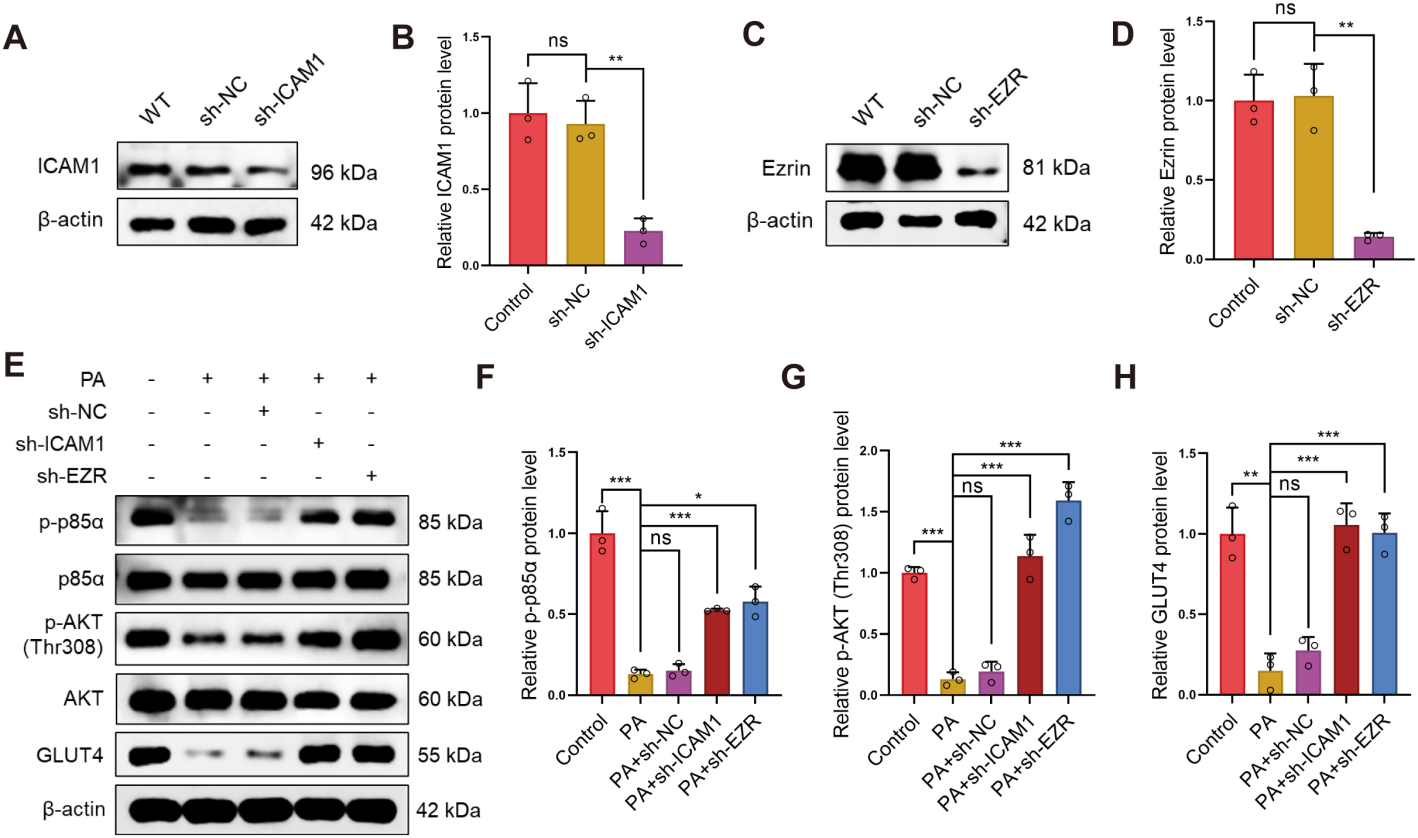
Knockdown of Ezrin and ICAM1 improves insulin signaling in PA-treated HepG2 cells **(A-D)** HepG2 cells were transfected with pLV3-U6-ICAM1 (human)-shRNA, pLV3-U6-EZR (human)-shRNA and scrambled control (sh-NC) respectively, and the knockdown expression of ICAM1 or EZR in HepG2 cells was validated using the western blot analysis. **(E-H)** Protein expression levels of p-p85α, p85α, p-AKT (Thr308), AKT, and GLUT4 were detected by western blot. The data are expressed as mean ± SD. n = 3 per group. ns, not significant; *p < 0.05, **p < 0.01, ***p < 0.001 *vs* control group.

In order to investigate the effects of ICAM1 and Ezrin on the insulin signaling pathway in HepG2 cells, we constructed low-expression cell lines for ICAM1 and Ezrin. Following cell transfection, the expression levels of ICAM1 and Ezrin in HepG2 cells were significantly reduced through the transfection of low-expression plasmids (**Figures 9A–D**). Previous research reports have indicated that the PI3K/AKT signaling pathway plays a crucial role in regulating various metabolic processes, including gluconeogenesis, insulin secretion, and lipid metabolism, with its dysregulation being associated with insulin resistance in T2DM (39, 40). Given the observed upregulation of ICAM1 and Ezrin in insulin resistant conditions, we hypothesized that these molecules may be associated with altered PI3K/AKT signaling in this cellular model. We observed that in the PA-induced HepG2 insulin resistance model, the knockdown of ICAM1 and Ezrin resulted in an elevated expression level of GLUT4 and improved glucose transport efficiency (**Figures 9E, H**). Furthermore, the inhibition of ICAM1 and Ezrin increased the levels of p-p85α and p-AKT (Thr308) (**Figures 9E–G**). These findings suggest that ICAM1 and Ezrin may be associated with impaired insulin signaling in the PA-induced HepG2 insulin resistance model, and their knockdown may partially restore PI3K/AKT pathway activation and glucose transport related protein expression.

### Ezrin and ICAM1 are significantly upregulated in the liver tissue of mice with T2DM

**Figure 10:**
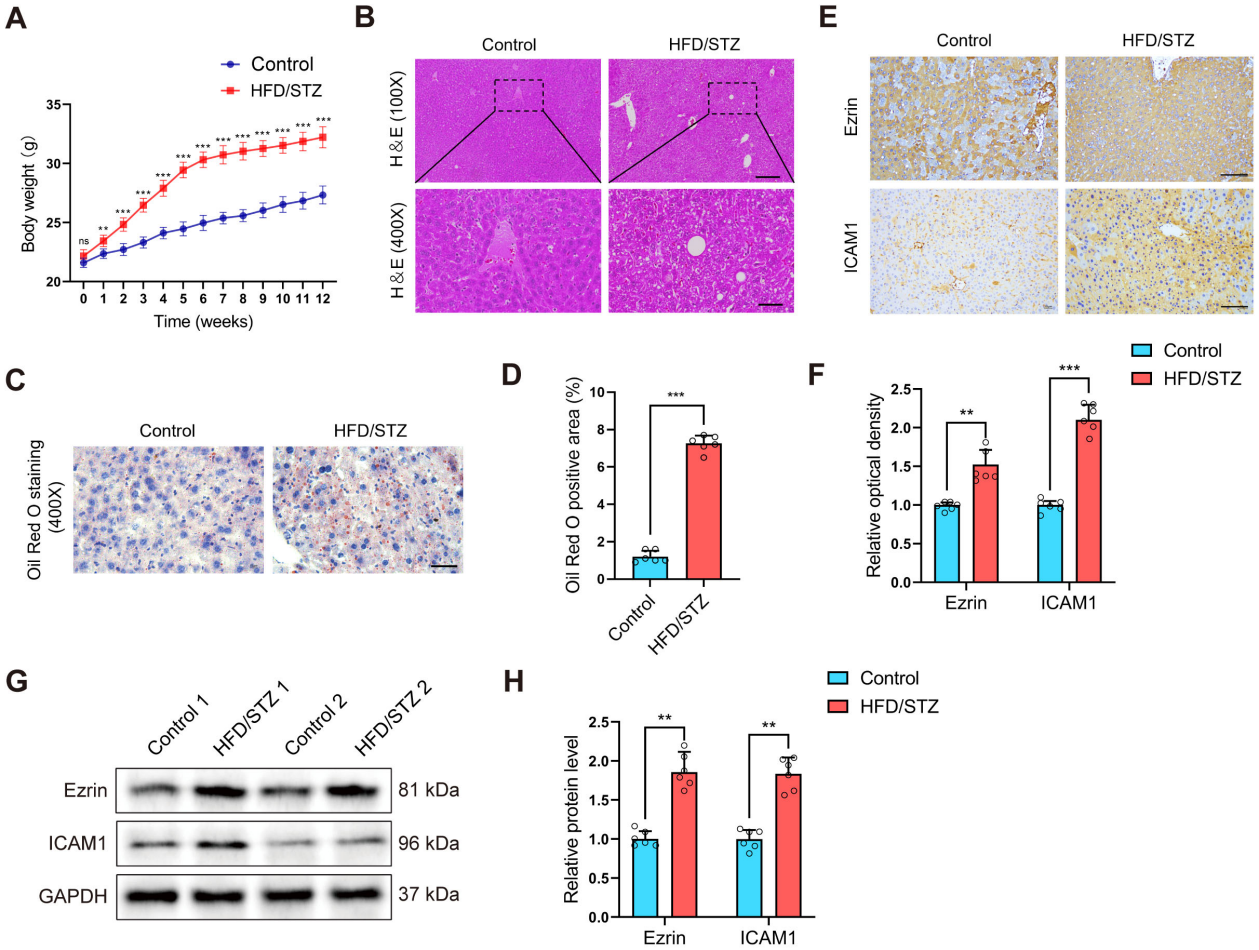
Ezrin and ICAM1 are significantly upregulated in the liver tissue of mice with T2DM. **(A)** Weight analysis of mice subjected to normal diet and HFD with STZ induction. **(B)** H&E staining images of mouse livers subjected to normal diet and HFD with STZ induction. Scale bar, 200 μm and 50 μm. **(C)** Oil Red O staining displays images of lipid droplet accumulation in the livers of mice subjected to a normal diet and a HFD, along with STZ induction. Scale bar, 50 μm. **(D)** Statistical analysis of the positive area stained by Oil Red (O) **(E)** IHC detection of the expression of Ezrin and ICAM1 in the livers of mice from each group. Scale bar, 50 μm. **(F)** Statistical analysis of the relative optical density values of Ezrin and ICAM1 in IHC. **(G)** Detection of the expression levels of Ezrin and ICAM1 in the liver of mice from each group using western blot analysis. **(H)** Statistical analysis of the expression levels of Ezrin and ICAM1. The data are expressed as mean ± SD. n = 6 per group. **p < 0.01, ***p < 0.001 *vs* control group.

To establish a model of T2DM, mice were subjected to a HFD for 12 weeks and received continuous injections of STZ for 5 days starting from the sixth week. Compared to the control group, the mice in the HFD/STZ group exhibited a significant increase in body weight (**Figure 10A**). H&E staining revealed significant structural disorganization of hepatocytes, an increase in cytoplasmic vacuolar changes, accompanied by varying degrees of histological damage (**Figure 10B**). The results of Oil Red O staining of liver tissue indicated that, compared to the control group, the HFD/STZ group exhibited a significant increase in lipid accumulation in the liver (**Figures 10C, D**). Subsequently, the immunohistochemical results of the liver indicated that the expression levels of Ezrin and ICAM1 in the HFD/STZ group were significantly elevated compared to the control group (**Figures 10E, F**). To further confirm the changes in the expression levels of Ezrin and ICAM1 in the liver of T2DM mice, we conducted western blot experiments to validate the expression levels of these two proteins. The results demonstrated that the expression levels of Ezrin and ICAM1 were significantly elevated in both HFD/STZ groups compared to the two control groups (**Figures 10G, H**).

## Discussion

T2DM is a multifaceted metabolic disorder that involves dysfunction of pancreatic β-cells and resistance to insulin (41). Insulin resistance, a key pathological feature of various metabolic disorders, is characterized by a diminished responsiveness of insulin-target tissues to physiological concentrations of insulin (42). Membrane proteins are involved in cellular signal transduction (43), substance transport (44, 45), cell adhesion (46, 47), and other processes, playing a crucial role in modulating insulin signaling pathways and maintaining metabolic homeostasis (48–50). However, the specific roles of membrane protein-related genes in the progression of T2DM remain poorly understood.

An expanding body of evidence suggests that membrane proteins play a critical role in diseases associated with insulin resistance. Proteins that interact with the cell membrane, such as solute carrier family 4 member 4 (SLC4A4), ATPase Na+/K+ transporting subunit beta 3 (ATP1B3), and glypican-4 (GPC4), in pancreatic β-cells and neuronal cells, attenuate GLP-1-induced insulin release by inhibiting the GLP-1 receptor (GLP-1R)-mediated cAMP signaling pathway (51). Monosaccharides produced by gut microbiota metabolism enhance the host’s intestinal absorption of monosaccharides through membrane transport proteins, such as the sodium-glucose cotransporter 1 (SGLT1) and glucose transporter (GLUT) families. This process can lead to insulin resistance (52). Furthermore, natural dietary polysaccharides improve hepatic glucose metabolism by activating the IR downstream insulin receptor substrate 1 (IRS1)/phosphatidylinositol 3-kinase (PI3K)/protein kinase B (AKT) signaling pathway (53). These findings highlight the crucial role of membrane proteins in regulating glucose metabolism. In this study, we utilized bioinformatics and machine learning techniques to examine the role of membrane protein-related genes in T2DM. Targeting the functional state of membrane proteins may offer new therapeutic strategies for treating insulin resistance-related diseases, such as T2DM, and has the potential to serve as novel therapeutic targets for insulin resistance.

In this study, ICAM1 and EZR were identified as membrane protein-related key genes associated with T2DM through integrated bioinformatics and machine learning analyses. Their diagnostic value was supported by nomogram and ROC curve analyses in external validation datasets. Functional enrichment analysis suggested that ICAM1/EZR-associated genes were mainly involved in immune regulation, leukocyte transendothelial migration, cell adhesion, actin cytoskeleton remodeling, and natural killer cell-mediated cytotoxicity, indicating that inflammatory responses and cytoskeletal organization may contribute to T2DM progression. Experimental validation further confirmed that ICAM1 and Ezrin were upregulated in insulin resistance models and in liver tissues of T2DM mice. Knockdown of ICAM1 or Ezrin activated PI3K-AKT signaling and increased GLUT4 expression in HepG2 cells, suggesting that these genes may be associated with impaired glucose transport and altered insulin signaling. Therefore, ICAM1 and EZR may represent potential diagnostic biomarkers and regulatory molecules involved in T2DM-associated insulin resistance.

Ezrin is a multifunctional protein that interacts with cortical actin filaments and plays a crucial role in diverse biological processes, including cell migration, endocytosis, cell survival, signal transduction, and translational regulation. Its functions are tightly regulated by various signaling molecules through phosphorylation cycles and dynamic conformational changes (54). Recent research evidence suggests that Ezrin protein may be related to the cellular processes associated with insulin resistance. In a model of insulin resistance induced by the O-GlcNAcase inhibitor O-(2-acetamido-2-deoxy-D-glucopyranosylidene) amino-N-phenylcarbamate (PUGNAc) in mouse C2C12 myotube cells, Ezrin is upregulated, leading to the ubiquitination of insulin receptors and the initiation of protein endocytosis (55). This finding is consistent with our research results. Furthermore, Ezrin interacts with ICAM1 through a single transmembrane segment in its cytoplasmic domain, playing a critical role in the intermolecular communication between adhesion proteins and the actin cytoskeleton.

ICAM1 is a cell adhesion molecule (CAM) that belongs to the immunoglobulin superfamily. It plays a crucial role in mediating the firm adhesion of leukocytes to endothelial cells during various acute and chronic inflammatory diseases (56). The activation of the PI3K/AKT signaling pathway has been demonstrated to mitigate diabetes-induced inflammation. ICAM1 functions as a pro-inflammatory mediator downstream of the PI3K/AKT pathway, and research has shown that ICAM1 levels are elevated in the STZ-induced rat model of diabetes (57, 58). The downregulation of ICAM1 may be a significant factor in improving the progression of T2DM.

The PI3K/AKT pathway is crucial for the transmission of insulin signals, thereby facilitating glucose uptake, glycogen synthesis, and insulin sensitivity (59). Our results suggest that ICAM1 and Ezrin may be associated with reduced phosphorylation of p85α and AKT in the PA-induced HepG2 insulin resistance model. The suppression of ICAM1 and Ezrin expression can activate the PI3K/AKT signaling pathway and enhance glucose transport efficiency. This is consistent with findings in T2DM, where dysfunction of the PI3K/AKT signaling pathway has been associated with insulin resistance and β-cell dysfunction (60, 61). Furthermore, our Co-IP results indicate that there may be a potential interaction between Ezrin protein and INSR, suggesting that Ezrin protein might be involved in the regulation of insulin signaling pathway. These research findings suggest a potential association between ICAM1 and Ezrin in the impaired insulin signaling within the insulin resistance model.

This study identified ICAM1 and EZR as potential diagnostic biomarkers for type 2 diabetes and confirms their upregulation in insulin resistance models through *in vitro* and *in vivo* experiments. However, *in vivo* gene knockout experiments have not been conducted. Previous studies have shown that diets rich in sugar and fat can induce insulin resistance and adipose tissue inflammation in mice, leading to elevated expression levels of ICAM1 in adipose tissue, which can exacerbate liver damage in diet induced obese mice (62, 63). EZR plays a crucial role in cellular membrane and cytoskeletal organization, receptor-associated signaling, and inflammation regulation (64). Further *in vivo* studies are necessary to elucidate whether the inhibition of ICAM1 or EZR can restore the PI3K-AKT signaling pathway, improve glucose transport, and alleviate insulin resistance. Furthermore, the HFD/STZ model combines diet induced insulin resistance with streptozotocin induced β-cell damage, which only partially reflects the heterogeneity of human type 2 diabetes mellitus (65, 66). Although HepG2 cells are commonly employed in studies of hepatic insulin resistance, they are derived from cancer cells and may not fully replicate the metabolic characteristics and insulin responsiveness of primary hepatocytes (67, 68). Consequently, the findings obtained from HFD/STZ induced mice and PA treated HepG2 cells should be regarded as preliminary evidence, necessitating further validation of the role of ICAM1/EZR in T2DM related insulin resistance in primary hepatocytes, liver organoids, and animal models that more closely mimic physiological conditions. In addition, the cell experiments were performed with three independent biological replicates, and no formal *a priori* power calculation was conducted. Therefore, the experimental results should be interpreted as preliminary functional validation. Future studies with larger independent experimental replicates, prospective cohorts, and standardized statistical planning are needed to further confirm the robustness and reproducibility of these findings.

As membrane proteins represent significant targets in the development of small molecule drugs, the discovery of ICAM1 and EZR in this study may provide new avenues for targeted therapy in T2DM (69, 70). Future research could further explore small molecule inhibitors, peptide inhibitors, or other targeted therapeutics aimed at ICAM1, EZR, or their downstream signaling pathways, assessing their potential to restore the activity of the PI3K-AKT signaling pathway, promote GLUT4-mediated glucose transport, and enhance insulin sensitivity. Moreover, it is essential to integrate structure-guided drug design, high-throughput screening, and *in vivo* pharmacological validation to further evaluate the druggability and therapeutic potential of ICAM1 and EZR as drug targets for T2DM.

In summary, this study employed bioinformatics and machine learning methodologies to analyze differentially expressed genes in T2DM, encompassing functional enrichment, GMM, RF, SVM, PPI networks, ROC curve analysis, and immune infiltration. The expressions of ICAM1 and EZR were validated using RT-qPCR, western blotting, and immunofluorescence assays in both *in vitro* and *in vivo* experiments, corroborating the bioinformatics findings. This study offers novel insights into the pathogenesis of T2DM and contributes to the identification of potential therapeutic targets.

## Conclusion

This study identified ICAM1 and EZR as membrane protein related genes associated with T2DM through integrated bioinformatics and machine learning analyses. The diagnostic model based on ICAM1 and EZR showed favorable diagnostic performance. In addition, the upregulation of EZR and ICAM1 mRNA and Ezrin and ICAM1 protein was validated in insulin resistance models and in liver tissues from T2DM mice. Functional experiments in PA treated HepG2 cells suggested that ICAM1 and Ezrin may be associated with impaired PI3K/AKT signaling and reduced glucose transport efficiency. However, further validation in larger independent cohorts, primary hepatocytes, and *in vivo* loss of function models is required to confirm their diagnostic robustness and mechanistic relevance in T2DM.

## Data Availability

The datasets presented in this study can be found in online repositories. The names of the repository/repositories and accession number(s) can be found in the article/**Supplementary Material**.
